# Interleukin-32α downregulates the activity of the B-cell CLL/lymphoma 6 protein by inhibiting protein kinase Cε-dependent SUMO-2 modification

**DOI:** 10.18632/oncotarget.2364

**Published:** 2014-10-20

**Authors:** Yun Sun Park, Jeong-Woo Kang, Dong Hun Lee, Man Sub Kim, Yesol Bak, Young Yang, Hee Gu Lee, JinTae Hong, Do-Young Yoon

**Affiliations:** ^1^ Department of Bioscience and Biotechnology, Bio/Molecular Informatics Center, Konkuk University, Seoul, South Korea; ^2^ Research Center for Women's Disease, Department of Life Systems, Sookmyung Women's University, Seoul, South Korea; ^3^ Medical Genomics Research Center, Korea Research Institute of Bioscience and Biotechnology, Daejeon, South Korea; ^4^ College of Pharmacy and Medical Research Center, Chungbuk National University, Cheongju, South Korea

**Keywords:** Interleukin 32α, B-cell CLL/lymphoma 6, Small Ubiquitin-like Modifier-2

## Abstract

A proinflammatory cytokine IL-32 acts as an intracellular mediator. IL-32α interacts with many intracellular molecules, but there are no reports of interaction with a transcriptional repressor BCL6. In this study, we showed that PMA induces an interaction between IL-32α, PKCε, and BCL6, forming a trimer. To identify the mechanism of the interaction, we treated cells with various inhibitors. In HEK293 and THP-1 cell lines, treatment with a pan-PKC inhibitor, PKCε inhibitor, and PKCδ inhibitor decreased BCL6 and IL-32α protein expression. MAPK inhibitors and classical PKC inhibitor did not decrease PMA-induced BCL6 and IL-32α protein expression. Further, the pan-PKC inhibitor and PKCε inhibitor disrupted PMA-induced interaction between IL-32α and BCL6. These data demonstrate that the intracellular interaction between IL-32α and BCL6 is induced by PMA-activated PKCε. PMA induces post-translational modification of BCL6 by conjugation to SUMO-2, while IL-32α inhibits. PKCε inhibition eliminated PMA-induced SUMOylation of BCL6. Inhibition of BCL6 SUMOylation by IL-32α affected the cellular function and activity of the transcriptional repressor BCL6 in THP-1 cells. Thus, we showed that IL-32α is a negative regulator of the transcriptional repressor BCL6. IL-32α inhibits BCL6 SUMOylation by activating PKCε, resulting in the modulation of BCL6 target genes and cellular functions of BCL6.

## INTRODUCTION

Interleukin (IL)-32 was first identified as a proinflammatory cytokine in activated T and Natural killer (NK) cells, and was originally named NK4 [1]. Many reports have revealed that IL-32α is expressed in various inflammatory cells including peripheral blood mononuclear cells (PBMCs), monocytes, NK cells, and T cells, as well as in non-immune cells such as fibroblasts, keratinocytes, and endothelial cells in response to various stimuli [2-5]. The proinflammatory properties of IL-32 were first identified in 2005 [1]. IL-32 has since been associated with cancer, viral infection, and inflammatory diseases such as rheumatoid arthritis (RA), ankylosing spondylitis, chronic obstructive pulmonary disease (COPD), and graft-versus-host disease (GVHD) [6-11]. IL-32 has several isoforms including α, β, γ, δ, ε, ζ, η, and θ which was recently discovered by our group [12]. IL-32 is induced by phorbol 12-myristate 13-acetate (PMA) in various cells including immune cells, such as monocytes/macrophages, and endothelial cells [13-15]. Although it was first reported as a cytokine, recent reports have established a role for IL-32α in intracellular pathways [13, 16, 17]. Recent studies have focused on the interactions between IL-32 isoforms [12, 18] and other proteins and transcriptional regulators [13, 16]. We recently reported that IL-32α binds to STAT3 and PKCε to induce IL-6 production [13]. IL-32β binds to C/EBPα and PKCδ to induce IL-10 production [16]. IL-32 isoforms also interact with each other [12]; in particular, IL-32δ interacts with IL-32β to inhibit IL-32β from inducing IL-10 production [18].

Epigenetic mechanisms play crucial roles in several cellular functions, including DNA damage and repair, RNA transcription and processing, and protein stability control and degradation to fundamental cellular process [19, 20]. Epigenetic post-translational modifications such as phosphorylation, methylation, ubiquitination, and SUMOylation (covalent modification with a small ubiquitin-like modifier [SUMO] protein) regulate cellular gene expression [21]. Ubiquitins and SUMOs, ubiquitin-like proteins (Ubls), play an important role in epigenetic control of gene expression. Both proteins attach covalently to other proteins on lysine residues by an isopeptide bond [22, 23]. SUMOylation sometimes requires the consensus sequence ΨKxE/D (where Ψ is a large hydrophobic residue and x is any amino acid) around the target lysine, although this consensus motif is also present in non-SUMOylated proteins [24]. SUMO also binds other proteins containing the consensus SUMO-interacting motif (SIM) [25, 26]. There are 4 SUMO isoforms, namely, SUMO-1, -2, -3, and -4 [27-29]. SUMO-1 is the primary SUMO protein in human cells and is involved in regulating protein stability along with ubiquitin [20, 22, 23, 30]. SUMO-2 and SUMO-3 have similar functions in regulating cellular processes such as signal transduction [28, 31].

B-cell CLL/lymphoma 6 (BCL6), which is encoded by the *ZBTB27* gene, formerly known as LAZ3, is similar to the promyelocytic leukemia zinc finger (PLZF) protein [32]. BCL6 is a POK/ZBTB protein. POK/ZBTB family proteins have an N-terminal, conserved BTB/POZ domain that interacts with other proteins, and Krüppel type (C_2_H_2_) zinc-finger (ZnF) motifs in the C-terminus that interact with DNA in a sequence-specific manner. These motifs are required to repress the transcription of target genes. POK/ZBTB proteins regulate diverse biological processes, including development of specific lineages in the immune system, lymphoid development, and oncogenesis [33-35]. In some diffuse large B-cell lymphomas (DLBCL), BCL6 protein expression was positively correlated with the mRNA level of Yin Yang 1 (YY1). YY1 expression was associated with B-cell transformation and tumor progression in both Burkitt's lymphoma and DLBCL [36]. This study highlights the role of IL-32α in regulating activity of the transcriptional repressor of BCL6. In this study, we demonstrate that IL-32α inhibits the transcriptional repressor function of BCL6, which targets genes such as c-myc, cyclin D2, CCL-3 [35, 37], and IL-6 [38], by interacting with BCL6 and inducing its SUMOylation.

## RESULTS

### PMA stimulates an interaction between IL-32α, BCL6 and PKCε

**Figure 1:**
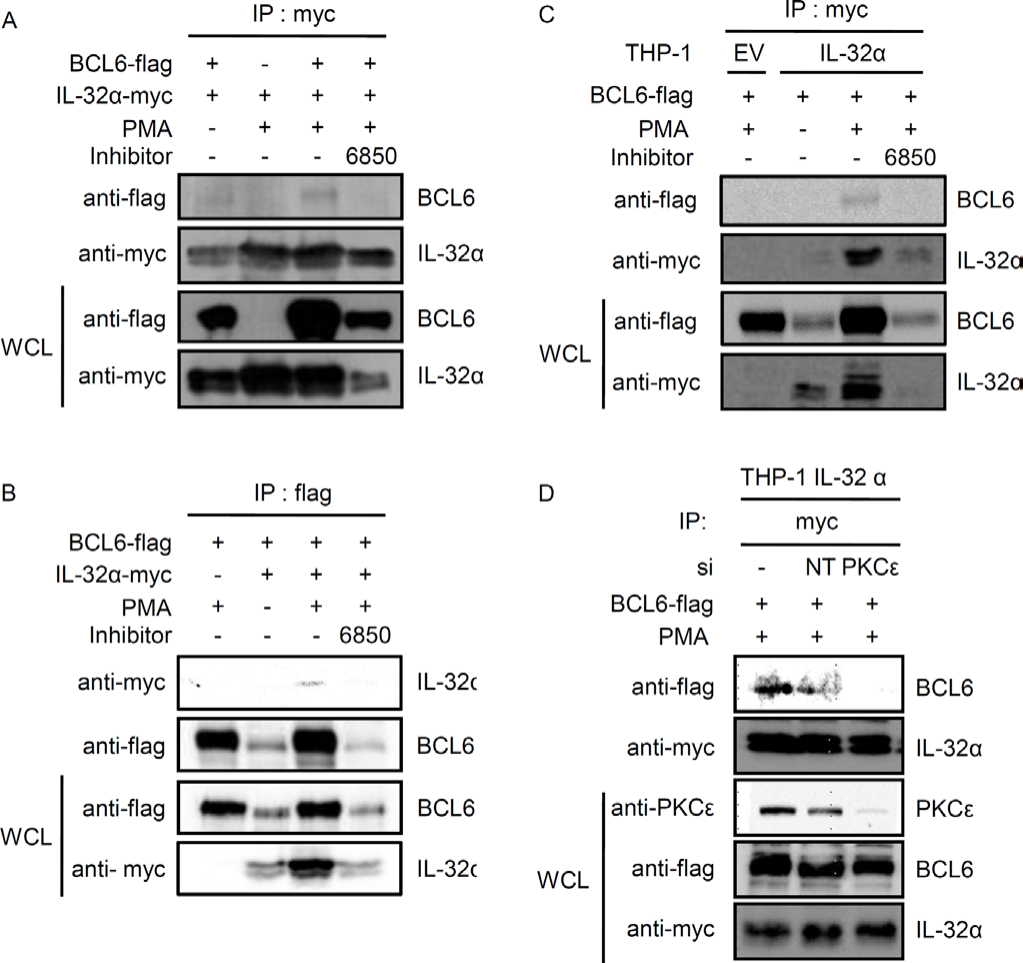
Interaction between IL-32α and BCL6 is mediated by PMA **(A and B)** HEK293 cells were cotransfected with a Myc-tagged–IL-32α expression vector and a FLAG-tagged-BCL6 expression vector. Twenty-four hours after transfection, the cells were pretreated for 2 h with 10 μM pan-PKC inhibitor, Gö6850 (6850), and then treated with PMA (50 nM) for an additional 3 h. Immunoprecipitation was carried out with 1 μg of myc tag antibody (A) or 2 μg of flag tag antibody (B) and 0.7 mg of whole cell lysate (WCL). Following transfection, IL-32α and BCL6 expression levels were assessed by western blot with 20 μg of WCL. **(C)** THP-1 EV and THP-1-IL-32α cells were transfected with a FLAG-tagged–BCL6 expression vector. After overnight incubation, cells were pretreated for 2 h with 10 μM pan-PKC inhibitor, Gö6850 (6850), and then treated with PMA (10 nM) for an additional 3 h. THP-1 cells lysates were prepared in the same way. Immunoprecipitation was carried out with 1 μg of myc tag antibody and 1 mg of WCL. **(D)** THP-1-IL-32α cells were co-transfected with a FLAG-tagged-BCL6 expression vector and 100 nM PKCε siRNA or nontargeting (NT) siRNA. These cells were treated with PMA (10 nM) for a additional 3 h. Immunoprecipitation was carried out in the same way, by using 1 μg of myc tag antibody. Precipitated BCL6 was detected with a flag tag antibody. *IP*, immunoprecipitation; *WCL*, whole cell lysate.

We recently observed the interaction between IL-32α and PLZF by using a yeast two-hybrid system (unpublished data). Because BCL6 is a member of the human BTB/POZ-zinc finger family-like PLZF and has a similar structure, we examined whether IL-32α also interacts with BCL6 [34, 39]. 6×Myc-tagged IL-32α and 5×FLAG-tagged BCL6 were cotransfected into HEK293 cells, followed by immunoprecipitation. Upon PMA stimulation, IL-32α interacts with BCL6. This interaction was diminished by treatment with the pan-PKC inhibitor Gö6850 (Fig. 1A and 1B). The interaction between IL-32α and BCL6 was further examined by immunoprecipitation in THP-1 EV and THP-1-IL-32α cells. The interaction between IL-32α and BCL6 was observed in THP-1-IL-32α cells stimulated with PMA, but not in the presence of Gö6850 (Fig. 1C). To investigate whether PKCε mediates the interaction between IL-32α and BCL6, we performed an immunoprecipitation assay after transfection with siPKCε. PKCε was almost completely knocked down by PKCε-specific siRNA relative to nontargeting siRNA. Following PKCε knockdown, the interaction between IL-32α and BCL6 was not observed after PMA treatment (Fig. 1D). These data suggest that IL-32α interacts with BCL6 when PKCε is activated by PMA.

**Figure 2:**
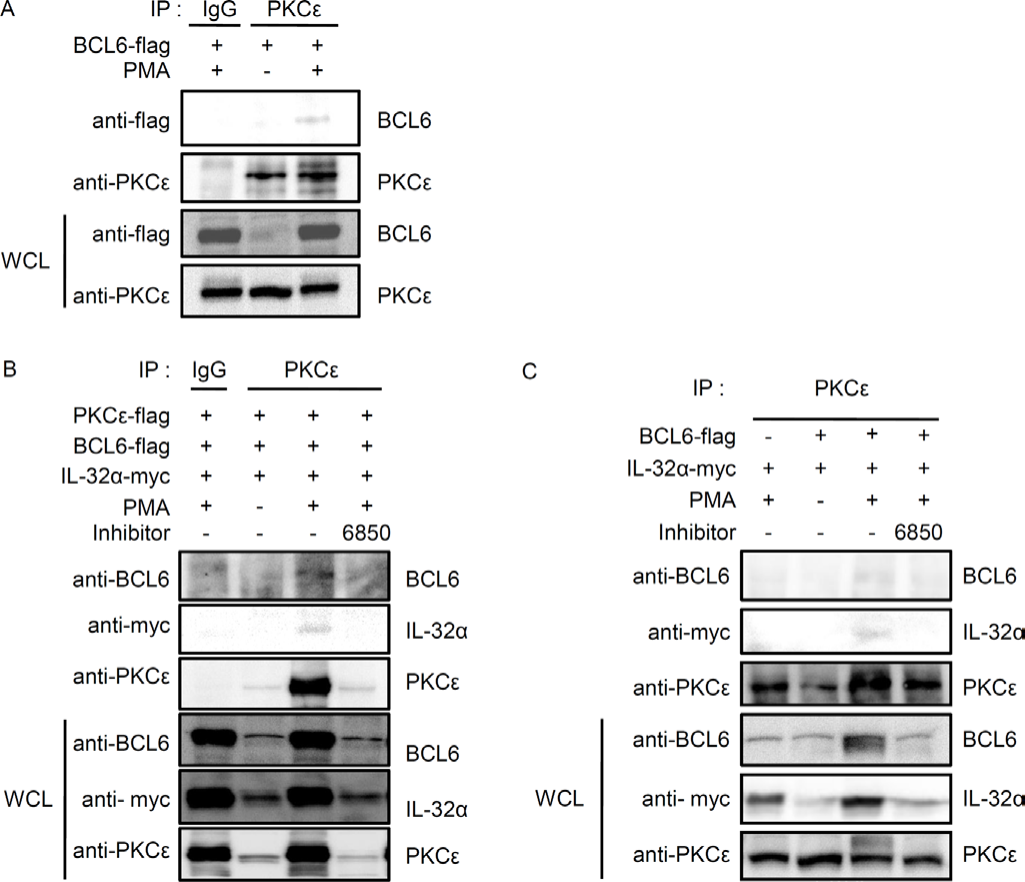
IL-32α interacts simultaneously with BCL6 and PKCε in the presence of PMA **(A)** HEK293 cells were transfected with FLAG-tagged BCL6. Cells were incubated overnight then treated with 50 nM PMA for 3h. **(B)** HEK293 cells were cotransfected with vectors expressing FLAG-tagged BCL6, FLAG-tagged PKCε, and Myc-tagged IL-32α. After cotransfection, HEK293 cells were treated with the pan-PKC inhibitor, Gö6850 (6850; 10 μM), for 2 h followed by 50 nM PMA for 6 h. **(C)** The interactions were also observed between endogenous PKCε, IL-32α, and BCL6. HEK293 cells were cotransfected with FLAG-tagged BCL6 and Myc-tagged IL-32α as indicated. HEK293 cells were pretreated with a pan-PKC inhibitor, Gö6850 (6850; 10 μM), for 2 h then treated with 50 nM PMA for 6 h. Immunoprecipitations with 1 μg of anti-PKCε or normal IgG antibody were performed, and the precipitated IL-32α and BCL6 was detected. Immunoprecipitation with normal rabbit IgG antibodies (IgG) was used as a control.

**Figure 3:**
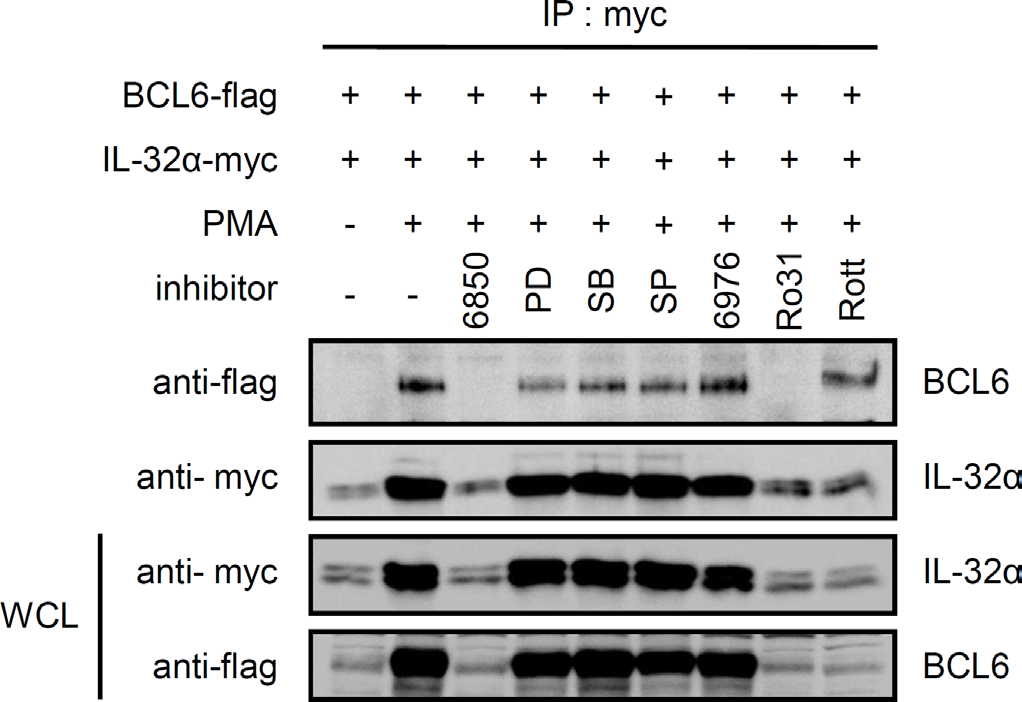
The interaction between IL-32α and BCL6 is mediated by PMA-activated PKCε HEK293 cells were cotransfected with a Myc-tagged IL-32α expression vector and FLAG-tagged BCL6. The transfected cells were pretreated with various inhibitors. Cells were treated with the pan-PKC inhibitor, Gö6850 (6850; 10 μM); ERK inhibitor, PD98059 (PD; 25 μM); p38 inhibitor, SB203580 (SB; 10 μM); classical PKC inhibitor, Gö6976 (6976; 10 μM); PKCε inhibitor, Ro-31-8220 (Ro31; 10 μM); and PKCδ inhibitor, Rottlerin (Rott; 10 μM), for 2 h, then with 50 nM PMA for an additional 3 h. Immunoprecipitation was performed with 1 μg of anti-myc tag antibody. Anti-flag antibody was used to detect BCL6 interacting with IL-32α.

We previously reported that IL-32α specifically interacts with PKCε and PKCδ [13]. Next, we explored whether BCL6 might also interact with PKCδ and PKCε. HEK293 cells were transfected with 5×FLAG-tagged BCL6, and immunoprecipitation was performed using normal IgG antibody (IgG) or anti-PKCε antibody. Endogenous PKCε interacted with BCL6 with PMA stimulation (Fig. 2A), while PKCδ did not (data not shown). We then examined whether IL-32α associated with BCL6 and PKCε together. To establish that IL-32α, BCL6, and PKCε interact simultaneously after PMA stimulation, we cotransfected cells with IL-32α, BCL6, and PKCε and performed immunoprecipitation. After immunoprecipitation with an anti-PKCε antibody, we detected the expression of both IL-32α and BCL6. These interactions were inhibited by treatment with Gö6850 (6850) (Fig. 2B). These interactions were also observed with endogenous PKCε (Fig. 2C). Taken together, these results suggest that PMA-stimulated PKCε enhances the interaction between IL-32α and BCL6 by forming a trimeric complex. Previous reports have shown that the IL-32α signaling pathway is mediated by the NF-κB and p38 MAPK signaling pathways [1]. Next we investigated whether MAPK and various PKC signaling pathways meditate the interaction between IL-32α and BCL6. HEK293 cells were transfected with 6× Myc-tagged IL-32α and 5×FLAG-tagged BCL6 and pretreated with various signaling pathway inhibitors before PMA activation. Treatment with inhibitors PD98059, SB203580, and SP600125 for ERK, p38, and JNK, respectively slightly decreased the interaction compared to PMA only controls (Fig. 3). Gö6850, a pan-PKC inhibitor, and Ro-31-8220, a PKCε-specific inhibitor, strongly inhibited the interaction between IL-32α and BCL6. Gö6976, a PKCα and β inhibitor, and Rottlerin, a PKCδ inhibitor, did not disrupt the interaction, although Rottlerin did decrease BCL6 and IL-32α expression (Fig. 3). These data imply that the interaction between IL-32α and BCL6 is specifically mediated by PMA-activated PKCε.

### IL-32α inhibits BCL6 SUMOylation conjugated by SUMO-2, and induces BCL6 ubiquitination upon PMA stimulation

**Figure 4:**
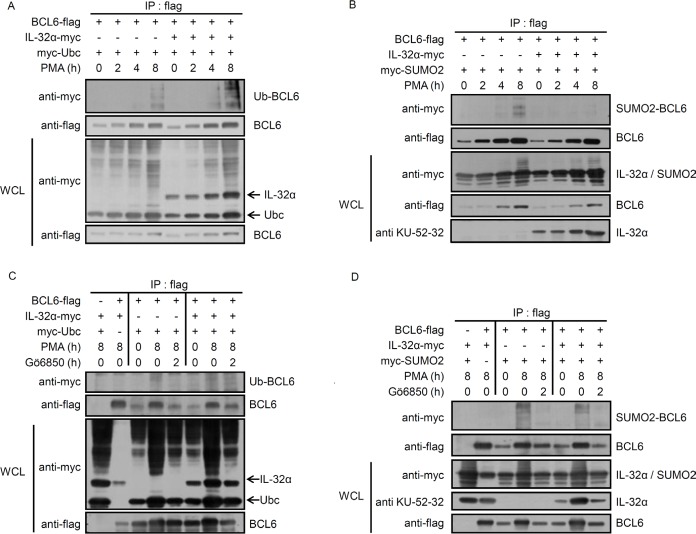

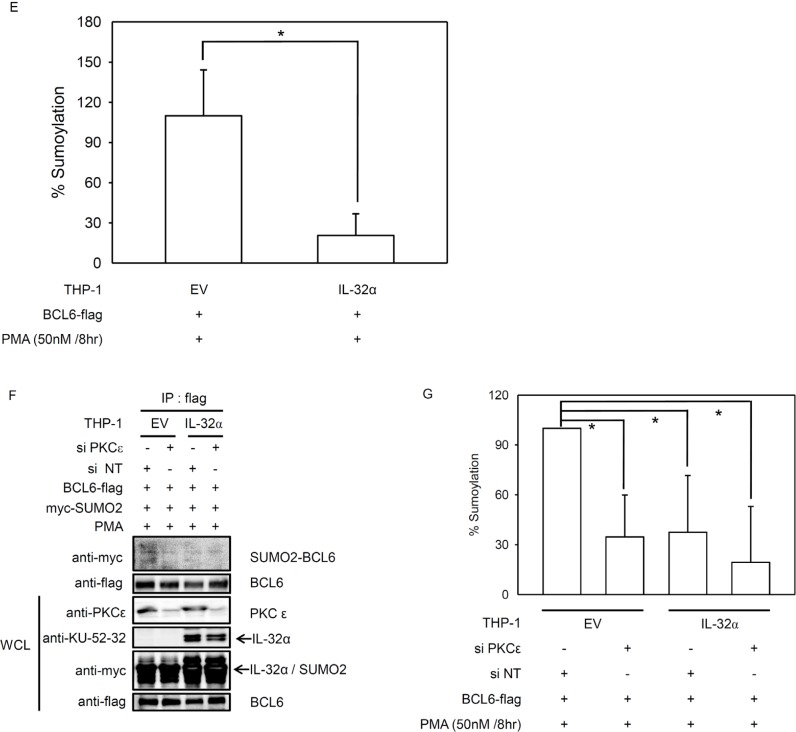
IL-32α mediates selection between ubiquitin or SUMO-2 conjugation to BCL6 **(A)** Expression constructs encoding Myc-tagged ubiquitin (2 μg) and FLAG-tagged BCL6 (1 μg) were cotransfected into HEK293 cells with or without the IL-32α expression vector (0.5 μg). After overnight incubation, transfected cells were incubated with PMA (50 nM) for the given time before harvest. Cell extracts were subjected to immunoprecipitation and immunoblotting carried out as described. Ubiquitin-modified BCL6 is indicated by trailing bands. **(B)** HEK293 cells were cotransfected with plasmids expressing Myc-tagged SUMO-2 (2 μg) and FLAG-tagged BCL6 (1 μg) in the presence or absence of an IL-32α expression plasmid (0.5 μg), and immunoprecipitation experiments were performed as described in (A). **(C and D)**, The experiments described in (A) and (B) were repeated, now using the pan-PKC inhibitor, Gö6850. Transfected cells were preincubated with pan PKC inhibitor, Gö6850 (6850; 10 μM) for 2 h followed by 50 nM PMA for 8 h. **(E)** THP-1-IL-32α and THP-1 EV were transfected with a construct expressing FLAG-tagged BCL6 with or without Myc-tagged SUMO-2 expression vector. After overnight incubation, THP-1 cells were treated with 50 nM PMA for 8 h, and then SUMO-2 conjugated BCL6 was detected in cell lysates with an *in vivo* SUMOylation ELISA, as described in Materials and Methods. **(F and G),** THP-1-IL-32α and THP-1 EV were transfected with a construct expressing FLAG-tagged BCL6, with or without a Myc-tagged SUMO-2 expression vector, and PKCε siRNA (siPKCε) or nontargeting siRNA (siNT) (100 nM). SUMOylation ELISA assay was performed in the same way. All data represent the mean ± SD of more than three independent experiments. *p<0.05.

Next, we investigated whether IL-32α mediates post-translational modifications of BCL6. Many studies indicate that the PLZF protein undergoes epigenetic regulation by SUMO-1 and ubiquitin conjugation. These modifications are functionally complementary [30]. Ubiquitination inhibits the transcriptional regulator PLZF through degradation and decreased protein stability [40], whereas SUMOylation upregulates PLZF by enhancing protein stability [41, 42]. BCL6 is a transcriptional repressor in the POK (POZ and Krüppel)/ZBTB (zinc finger and BTB) protein family, along with PLZF. BCL6 is structurally and functionally similar to PLZF [34]. Ubiquitination of BCL6 has been reported to affect its transcriptional repressor activity [35, 43, 44]. We investigated whether the interaction between BCL6 and IL-32α regulates BCL6 ubiquitination. We cotransfected HEK293 cells with BCL6 and ubiquitin along with IL-32α or empty vector and treated the cells with PMA for various times. Whole cell lysates were immunoprecipitated with anti-flag antibody to pull down BCL6. As shown in Figs. 4A, and 4C, IL-32α had no effect on BCL6 protein expression. However, PMA treatment did induce BCL6 ubiquitination in IL-32α expressing cells, compared to empty vector cells (Fig. 4A). BCL6 ubiquitination was suppressed by Gö6850 (Fig. 4C). These data indicate that IL-32α induces ubiquitination of BCL6 with PMA activation of PKC. SUMO-1 enhances PLZF stability by competitively binding at ubiquitin-binding sites [30]. The ZBTB1 protein contains an amino-terminal BTB domain and eight zinc fingers and is conjugated by SUMO-2 [45]. We observed that IL-32α induces SUMO-2 conjugation to PLZF in response to PMA stimulation (unpublished data). BCL6 ubiquitination is known to lead to protein degradation. IL-32α, however, does not alter BCL6 protein expression (Figs. 1C, 4A, and 4C). Therefore, we investigated the possible interaction between SUMO-2 and BCL6. PMA treatment for 8 h induced SUMO-2 modification of BCL6, whereas SUMOylation was inhibited in IL-32α-expressing cells (Fig. 4B). Because Gö6850 inhibited SUMO-2 modification, SUMOylation of BCL6 is PKC dependent in nature (Fig. 4D). These results imply that IL-32α, mediated by PKC, inhibits BCL6 interaction with SUMO-2. The specificity of BCL6 modification by SUMO-2 was further verified with quantitative SUMOylation assays in THP-1 cells. THP-1 EV and THP-1-IL-32α cells were cotransfected with SUMO-2 and BCL6 and treated with PMA for 8 h. SUMO-2 interaction with BCL6 was detected by an ELISA assay. IL-32α significantly inhibited SUMO-2 conjugation to BCL6. This result (Fig. 4E) confirmed the results of the immunoprecipitation assay (Fig. 4B). To further assess the effects of PKCε on SUMOylation of BCL6, we performed immunoprecipitation and quantitative SUMOylation assays using RNAi to specifically downregulate PKCε. When siPKCε was transfected into THP-1 EV and THP-1-IL-32α cells, the percentage of SUMOylated BCL6 was decreased in THP-1 EV cell lines (Figs. 4F and G). Thus, these data indicate that IL-32α regulates SUMO-2 modification of BCL6 via PKCε signaling. Figs. 3 and 4 show that PKCε can mediate both BCL6 ubiquitination and SUMOylation. These post-translational modifications are regulated by IL-32α in a functionally complementary manner.

### IL-32α drives IL-6 production and inhibits the transcriptional repressor BCL6, upregulating BCL6 target gene expression

**Figure 5:**
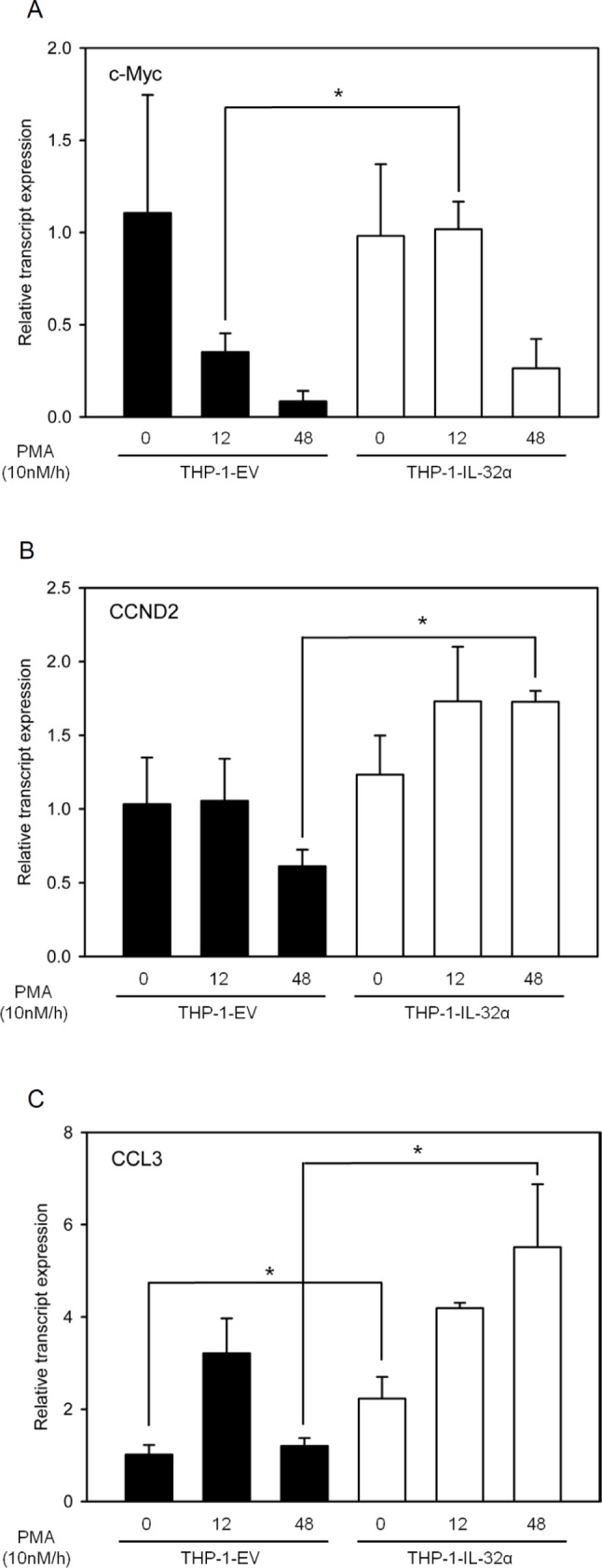
IL-32α is a potent inhibitor of the transcriptional repressor BCL6 THP-1 EV cells and THP-1-IL-32α cells were transfected with FLAG-tagged BCL6 plasmid, and then incubated overnight. Transfected cells were treated with 10 nM PMA and harvested at the indicated time points. Total RNAs were extracted, and the expression levels of several cell-signaling–related genes that are suppressed by BCL6 were assessed by real-time PCR. c-Myc **(A)**, CCND2 **(B)**, and CCL3 **(C)**. c-Myc is the v-myc avian myelocytomatosis viral oncogene homolog, CCND2 is cyclin D2, and CCL3 is chemokine (C-C motif) ligand 3. GAPDH levels were used for normalization. Results are means ± SEM of 3 independent experiments. Statistical significance (*, *p* < 0.05) was determined by t-test.

To assess the effects of IL-32α-mediated post-translational modification of BCL6, we evaluated the expression of BCL6 target genes. THP-1 cell lines stably expressing either IL-32α or empty vector were transfected with BCL6 and then incubated with PMA for 0, 12, or 48 h. qRT-PCR was performed to assess the expression of well-known BCL6 target genes, namely, c-Myc, cyclin D2, and CCL3 [37, 46-48]. THP-1 cells expressing IL-32α had increased c-Myc expression compared to the empty vector. Increased c-Myc expression was maintained at 12 h, decreased by 48 h, more stable than in THP-1 EV cells, where the expression levels decreased considerably between 12 h and 48 h (Fig. 5A). Cyclin D2 and CCL3 expression increased significantly in the presence of BCL6 at 48 h in THP-1-IL-32α cells compared to THP-1 EV cells (Figs. 5B and 5C). Increased c-Myc, cyclin D2, and CCL3 gene expression may result from decreased BCL6 activity in response to PMA-activated IL-32α.

**Figure 6:**
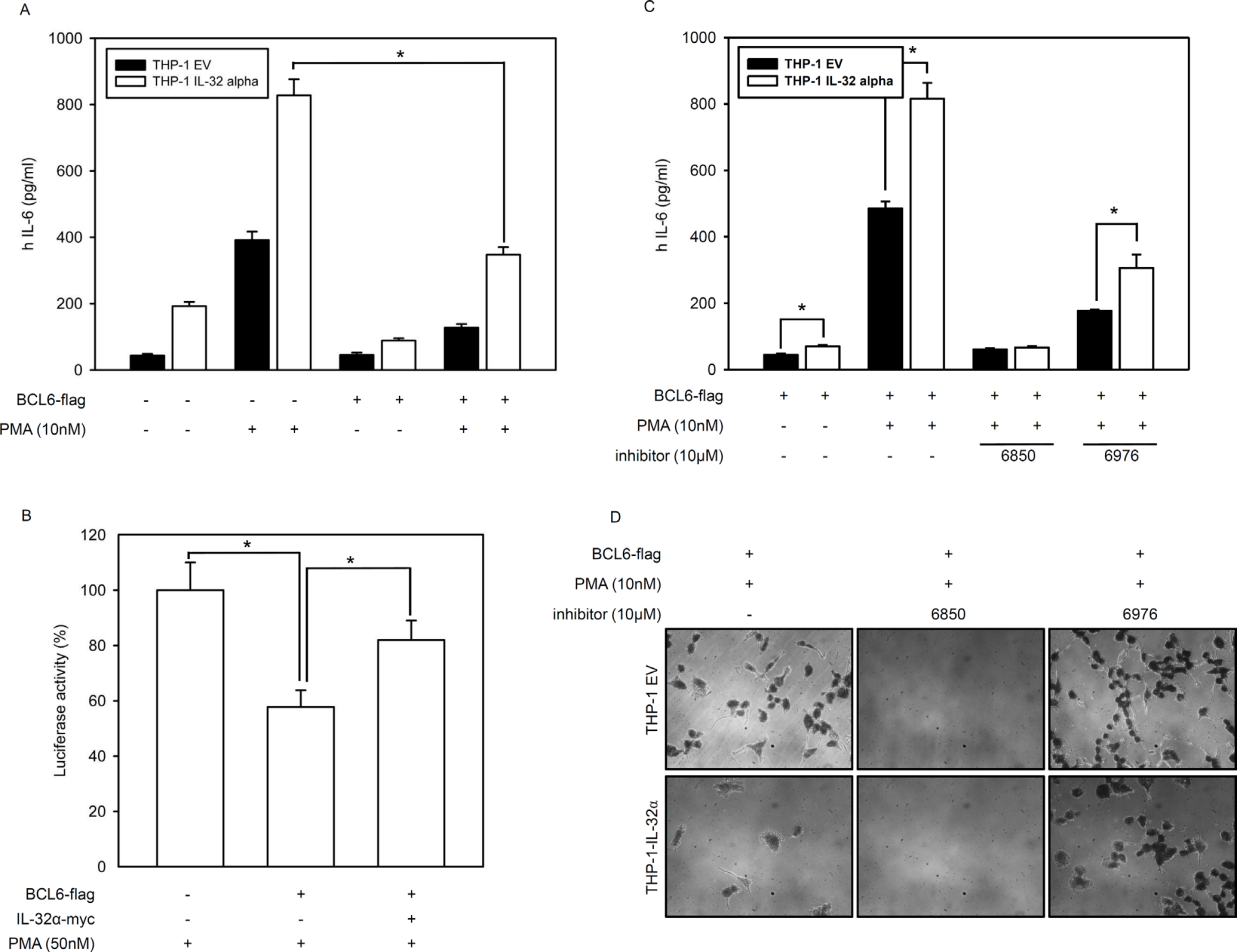
IL-32α induces IL-6 production by inhibiting BCL6 **(A)** FLAG-tagged BCL6 was transfected into THP-1 EV and THP-1-IL-32α cells as indicated. After overnight incubation, cells were treated with 10 nM PMA for 24 h. Culture supernatants were measured by ELISA for IL-6 secretion. **(B)** HEK293 cells were transfected with vectors for an IL-6 promoter-firefly luciferase reporter plasmid (as previously reported [13], 0.5 μg) and a plasmid expressing PKCε (1 μg) in the absence or presence of a BCL6 vector (1 μg) and IL-32α-expressing vector (1 μg), as described. After overnight incubation, cells were further incubated with 50 nM PMA for 24 h. The cell lysate was then subjected to a dual-luciferase assay. Results are presented as percent activity relative to the activity in cells transfected with neither IL-32α nor BCL6 (set a 100%). Data (mean and SEM.) are representative of at least six independent experiments. **(C)** THP-1 EV and THP-1-IL-32α cells were transfected with BCL6. These cells were pretreated with Gö6850 (6850; 10 μM) or Gö6976 (6976; 10 μM) for 2 h. followed by treatment with PMA of 10nM for 24 h. **(D)** BCL6 expressing THP-1 EV and THP-1-IL-32α cells were pretreated with the inhibitors Gö6850 (6850; 10 μM) or Gö6976 (6976; 10 μM) for 2 h. and incubated for another 48 h with 10 nM PMA. Cell adhesion and morphology were analyzed using phase-contrast microscopy.

We previously reported that IL-32α induces STAT3 phosphorylation and IL-6 production in THP-1 human monocytic cells in response to PMA treatment [13]. Macrophages from BCL6 -/- mice produce significantly more IL-6 than wild-type macrophages [38]. To evaluate the production of IL-6 with BCL6 inhibition by IL-32α, we transfected THP-1-IL-32α and THP-1 EV cells with BCL6 and treated them with PMA. ELISA revealed that PMA significantly increased IL-6 production in both cell lines, but with greater magnitude in the presence of IL-32α, as previously reported [13]. Compared to the cells transfected with empty vector, BCL6-transfected cells produced considerably less IL-6. IL-32α induced IL-6 production was suppressed by BCL6. These results strongly suggest that PMA activates endogenous IL-32α expression in THP-1-IL-32α cells, which therein recovers the IL-6 production suppressed by BCL6 (Fig. 6A). To investigate this further, we examined how IL-32α suppressing BCL6 might affect the activity of the IL-6 promoter in HEK293 cells cotransfected with PKCε and the IL-6 promoter. IL-6 reporter activity was suppressed to almost 57% in the presence of BCL6. When IL-32α was cotransfected along with PKCε and the IL-6 promoter, the reporter activity fully recovered (Fig. 6B). So far, our results demonstrate that IL-32α negatively regulates the effects of BCL6 on BCL6 target gene expression and IL-6 production. We then explored whether PKCε mediates this inhibitory activity of IL-32α with respect to the cellular functions of BCL6. THP-1 EV and THP-1-IL-32α cells were transfected with BCL6. These cells were pretreated with Gö6850 (6850) or Gö6976 (6976) before PMA activation. On treatment with Gö6850, IL-6 production was completely inhibited in both cell lines. Treatment with the classical PKC inhibitor, Gö6976 also decreased IL-6 production compared to treatment with PMA alone, but the fold-increase ranges noted for THP-1-IL-32α cells were similar to those observed after PMA treatment, compared to THP-1 EV cells (Fig. 6C). The adhesion of THP-1 cells with PMA was inhibited in the presence of IL-32α, as previously reported [17]. BCL6 gene expression is continuously increased in PMA-stimulated THP-1 monocyte-macrophage differentiation [49]. To verify that PKCε is involved in the adhesion of THP-1 cells, we treated them with PKC inhibitors. Pan-PKC inhibitor Gö6850 perfectly inhibited adhesion in both cells lines. However, cells treated with the classical PKC inhibitor Gö6976 showed that the inhibitory effect on the adhesion induced by IL-32α might be attributed to the non-classical PKCs (Fig. 6D). These results demonstrate that IL-32α regulates the function of BCL6 via IL-6 production and cell adhesion through PKCε mediation.

## DISCUSSION

IL-32 has been described as a proinflammatory cytokine. Its structure is unique among cytokines, and no specific cell-surface receptor of IL-32 has been identified. Many studies have focused on the biological functions of IL-32 exerted through interactions with intracellular molecules [13, 16, 18, 50]. Using a yeast two-hybrid system to screen IL-32α interacting proteins, we identified PLZF. We predicted that BCL6 also interacts with IL-32α because BCL6, like PLZF, is also a POK/ZBTB protein [32, 34]. BCL6 and PLZF have many analogous properties which include molecular structure and cellular functions. IL-32α induces IL-6 production by interacting with PKCε and STAT3 upon PMA treatment [13]. BCL6 represses IL-6 production. Macrophages from BCL6 (-/-) mice produce more IL-6 than do wild-type macrophages [38]. Our results show that IL-32α interacted with BCL6 after PMA stimulation. This interaction was diminished by pretreatment with a pan-PKC inhibitor. Therefore, we sought to determine if PKCε mediates this interaction by PMA. THP-1-IL-32α cells were transfected with siPKCε to lack PKCε, and the results of immunoprecipitation eliminates the possibility of an interaction between IL-32α and BCL6 (Fig. 1). We investigated whether BCL6 interacts with PKCδ and PKCε because IL-32α interacts with both PKCs [13]. We found that BCL6 did not interact with PKCδ (data not shown). We also demonstrated that IL-32α, BCL6, and PKCε form a trimeric complex (Fig. 2). Immunoprecipitation analyses using inhibitors showed that PKCε is the primary signaling molecule in the interaction between IL-32α and BCL6. MAPKs, NF-κB, and other PKCs had only a minor effect (Fig. 3). These results support that IL-32α acts as a modulator by interacting with PKCε [13].

PLZF is modified by ubiquitin and SUMO-1, and these modifications regulate its biological function. Ubiquitin and SUMO-1 conjugation are competitive. Furthermore, the transrepression activity and stability of PLZF are determined by the antagonistic relationship between these two post-translational modifications [30, 41, 42]. BCL6 functions as a transcriptional repressor by interacting with various corepressors to regulate the cell cycle and immune responses [37, 51-54]. BCL6 ubiquitination has already been reported [35, 43]. BCL6 protein is phosphorylated by MAPKs, ERK-1 and ERK-2, which leads to BCL6 degradation via the ubiquitin/proteasome pathway [44]. We observed that PMA treatment resulted in more BCL6 ubiquitination in the presence of IL-32α than in the absence of IL-32α (Fig. 4). IL-32α had no effect on BCL6 protein expression or localization (data not shown), while PMA enhanced BCL6 expression in the presence or absence of IL-32α. This result implies that IL-32α mediated ubiquitination does not affect BCL6 degradation. To further elucidate the mechanism by which IL-32α regulates BCL6 modification, we next evaluated SUMO-2 modification of BCL6. We found for the first time that BCL6 was modified by SUMO-2. PMA-mediated BCL6 SUMOylation was inhibited by IL-32α. This modification was dependent on PKCε activation, because Gö6850 and siPKCε inhibited the BCL6 SUMOylation (Fig. 4). These results indicate that reduction of SUMO-2-conjugated BCL6 by IL-32α was mediated by PKCε. Previous reports indicate that blocking BCL6 induces the expression of cell cycle-related genes [37, 55]. Assuming that IL-32α is related to the cell cycle by repressing BCL6, we analyzed several cell cycle profiles by qRT-PCR. IL-32α induced the expression of several cell cycle genes (Fig. 5). These data suggest that IL-32α is involved in inhibiting differentiation and enhancing proliferation by inhibiting BCL6. This result is also supported by our previous reports that IL-32α significantly suppresses differentiation into macrophage-like cells by inhibiting cell adhesion molecules on THP-1 cells [17]. IL-32 has been studied in inflammatory responses. Several studies reported that IL-32β and IL-32γ induce production of the anti-inflammatory cytokine IL-10 [16, 18, 56, 57], and IL-32α, in particular, promotes IL-6 production in myeloid cells [13]. IL-6 is a transcriptional target of BCL6. BCL6 negatively regulates IL-6 production by specifically binding to consensus sites on IL-6 [38]. Consistent with previous reports, IL-32α induced IL-6 production. In addition, BCL6-induced downregulation of IL-6 was recovered in the presence of IL-32α. We demonstrate that the mechanism of IL-6 production regulated by the interaction of IL-32α and BCL6 was mediated PKCε. To clarify a cellular function of IL-32α, we performed cell adhesion assay (Fig. 6). In keeping with results of previous reports, our data suggest that inhibition of cell adhesion by IL-32 was mediated by PKCε [17]. Before this study, limited information existed on the role of IL-32α in intracellular signaling. A study of the interaction between IL-32α and target proteins suggested that an immune response regulated the effect, although the specific receptors and a detailed mechanism remained unclear. Our work demonstrates a role for IL-32α as an intracellular modulator in monocytes by acting as an inhibitor of a potent transcriptional regulator, BCL6.

## MATERIALS AND METHODS

### Reagents and cell culture

Human promyelomonocytic THP-1 and human embryonic kidney (HEK) 293 cells were purchased from American Type Culture Collection (ATCC, Rockville, MD). THP-1 cells were grown in RPMI 1640 (WelGENE, Daegu, Korea) supplemented with 2 mM l-glutamine, 100 U/ml penicillin, 100 μg/ml streptomycin, and 10% fetal bovine serum (FBS; Hyclone, Logan, UT). HEK293 cells were grown in Dulbecco's modified Eagle's medium (DMEM) (Invitrogen, Seoul, Korea) containing 10% fetal bovine serum (FBS; Hyclone Laboratories, Logan, UT). Phorbol 12-myristate 13- acetate (PMA) was purchased from Sigma (Sigma, St. Louis, MO). MAPK inhibitors (PD98059, SB203580, and SP100625) and PKC inhibitors (Gö6850, Gö6976, Ro-31-8220, and Rottlerin) were purchased from Calbiochem (Calbiochem, San Diego, CA). The IL-32α-expressing THP-1 stable cell lines were previously established [13].

### Construction of expression vectors

IL-32α cDNA was subcloned into pcDNA3.1 + 6×Myc vector using *E*coRI and *X*hoI. cDNAs for PKCδ and PKCε were subcloned into the pcDNA3.1 + 5×FLAG vector using *E*coRI and *X*hoI [13]. BCL6, SUMO-2, and ubiquitin cDNAs were PCR-amplified from a human spleen cDNA library (Clontech, Palo Alto, CA). The primers sets were as follows: BCL6: sense 5′-GAA TTC ATG GCC TCG CCG GCT GAC AGC TG-3′, antisense 5′-CTC GAG GCA GGC TTT GGG GAG CTC CGG AG-3′; SUMO-2: sense 5′- GCT GAA TTC ATG GCC GAC GAA AAG CCC-3′, antisense 5′- GAA CTC GAG CTA ACC TCC CGT CTG CTG-3′; ubiquitin: sense 5′-GGA TCC ATG CAG ATC TTC GTG AAA ACC-3′, antisense 5′-CTC GAG CTA ACC ACC TCT CAG ACG CAG-3′. The entire BCL6 gene was amplified and cloned into pcDNA3.1 + 5×FLAG vector using *E*coRI and *X*hoI. SUMO-2 and ubiquitin cDNAs were subcloned into the pCS3MT + 6×Myc vector using *E*coRI and *X*hoI.

### Transfection

THP-1 promonocytic cells were transfected with pcDNA3.1 + 5×FLAG-BCL6 and pCS3MT + -SUMO-2 or -ubiquitin using the Neon™ transfection system (Invitrogen, Carlsbad, CA) according to the manufacturer's instructions. PKCε siRNA was transfected in the same way. PKCε siRNA and non-targeting siRNA were purchased from Mbiotech (Mbiotech, Hanam, Korea) according to a previously reported siPKCε sequence [58]. HEK293 cells were transfected with pcDNA3.1 + 6×Myc-IL-32α, pcDNA3.1 + 5×FLAG-BCL6 and pCS3MT + -SUMO-2 or -ubiquitin by using Lipofectamine 2000 (Invitrogen, Carlsbad, CA) according to the manufacturer's instructions.

### Western blot and immunoprecipitation

HEK293, THP-1 EV, and THP-1-IL-32α cells were cotransfected with pcDNA3.1 + 6×Myc-IL-32α, pcDNA3.1 + 5×FLAG-BCL6, and pCS3MT + -SUMO-2 or -ubiquitin and then lysed in 50 mM HEPES (pH 7.5), 150 mM NaCl, 5% glycerol, 20 mM β-glycerophosphate, 1% NP-40, 0.5% TX-100, and 1 mM EDTA. Western blot was performed with an anti-myc tag antibody (Millipore, Bedford, MA), anti-flag tag antibody (Sigma, St. Louis, MO), anti-PKCδ/ε antibody (Santa Cruz Biotechnology, TX), anti-IL-32 antibody KU32-52 [15, 17], and anti-BCL6 antibody (Santa Cruz Biotechnology). For immunoprecipitation, cell lysates were mixed with 1 μg of anti-myc tag antibody, 1 μg of anti-PKC δ/ε antibody, and 2 μg of anti-flag tag antibody for 1 h and then precipitated with 35 μl of protein G-agarose beads (KPL, Gaithersburg, MD).

### Reverse-transcription polymerase chain reaction (RT-PCR) and real-time qPCR analyses

THP-1 EV and THP-1-IL-32α cells were treated with 10 nM PMA for the indicated time, and total RNA was extracted for RT-PCR. Total RNA was extracted using the Easy-Blue total RNA extraction kit (iNtRon Biotechnology, Seoul, Korea). For cDNA synthesis, reverse transcription was performed with 2 μg of the total RNA, oligo dT, dNTPs, DTT, buffer and Superscript M-MuLV reverse transcriptase (New England Biolabs, Beverly, MA). cDNA was analyzed by real-time qPCR using iQ SYBR Green Supermix (both from BioRad, Hercules, CA) according to the manufacturer's instructions for relative quantification by using Chromo 4 (Bio-Rad, Hercules, CA). Target gene expressions were normalized to that of the housekeeping gene, GAPDH. Primers sets were as follows: c-Myc: sense 5′-TCA AGA GGC GAA CAC ACA AC-3′, antisense 5′-GGC CTT TTC ATT GTT TTC CA-3′; CCND2: sense 5′-CCG GAC CTA ATC CCT CAC TC-3′, antisense 5′-CAC ACC GAT GCA GCT TTC TA-3′; and CCL3: sense 5′-GGT CTC CAC TGC TGC CCT TGC-3′, antisense 5′-GGA ATC TGC CGG GAG GTG TAG C-3′.

### SUMOylation assay

We used a commercially available SUMOylation Assay Kit (Abcam, San Francisco, CA) according to the manufacturer's instructions. To summarize briefly, THP-1 EV and THP-1-IL-32α were cotransfected with pcDNA3.1 + 5×FLAG-BCL6, and pCS3MT + SUMO-2 and were then treated with 50 nM PMA for 8 h. Cells were lysed in 50 mM HEPES (pH 7.5), 150 mM NaCl, 5% glycerol, 20 mM β-glycerophosphate, 1% NP-40, 0.5% TX-100, and 1 mM EDTA. Extracted whole cells lysates were incubated with anti-flag tag antibody (0.2 μg/well) (Sigma. Louis, MO) for 60 min and then a SUMO detection antibody was added. After incubation, a signal reporter solution was added. Finally, a color developing solution was added and the absorbance was measured at 450 nm by a microplate reader. We calculated SUMOylation of the BCL6 protein as follows.

$$\%\;\text{SUMOylation}=\frac{\text{OD}\;(\text{SUMO}2\;\text{transfected}\;\text{sample}-\text{negative}\;\text{control})}{\text{OD}\;(\text{SUMO}2\;\text{untransfected}\;\text{sample}-\text{negative}\;\text{control})}\times 100\%$$

### Measurement of IL-6 production levels and IL-6 promoter activity

The IL-6 primer set was taken from a previous study [13]. pGL3-IL-6 promoter (0.5 μg), pRL-null (Renilla, 0.5 μg), and PKCε (1 μg) expression vectors were cotransfected into HEK293 cells with or without the IL-32α expression vector (1 μg) and BCL6 expression vector (1 μg). Luciferase assays were performed using the Dual-Luciferase® reporter assay system (Promega, Madison, WI). IL-6 ELISA was performed using an IL-6 ELISA kit (R&D Systems, Minneapolis, MN) according to the manufacturer's instructions.

### Cell morphology analysis

BCL6-induced THP-1 EV and THP-1-IL-32α cells were pretreated with inhibitors and incubated for 48 h with 10 nM PMA. Adherent or differentiated cells were analyzed by phase contrast microscopy. The cell morphology was assessed using REASTAIN QUICK-DIFF Kit (Reagena, Toivala, Finland) according to the previous studies [17].

### Statistical analysis

Data presented are the mean ± SEM of the results of at least three independent experiments. Statistical significance was assessed with Student's t-test. A *p-value* < 0.05 was considered to be statistically significant.

## Abbreviations:

PMA: phorbol 12-myristate 13-acetate
PKC: protein kinase C
IL-32: interleukin-32
BCL6: B-cell CLL/lymphoma 6
EV: empty vector
